# Mothers and not genes determine inherited differences in cadmium sensitivities within unexposed populations of the freshwater crustacean *Gammarus fossarum*

**DOI:** 10.1111/eva.12327

**Published:** 2015-10-28

**Authors:** Amandine Vigneron, Olivier Geffard, Hervé Quéau, Arnaud Chaumot

**Affiliations:** ^1^ IrsteaUR MALY Milieux aquatiques, écologie et pollutions, centre de Lyon‐VilleurbanneVilleurbanneFrance

**Keywords:** adaptation, contaminants, evolutionary ecotoxicology, gammarids, metals, quantitative genetics

## Abstract

Deciphering evolutionary processes occurring within contaminated populations is important for the ecological risk assessment of toxic chemicals. Whereas increased tolerance to contaminants is well documented in aquatic animal populations, whether such phenotypic changes occur through genetic adaptation is still debated. In that sense, several studies with the freshwater crustacean *Gammarus* concluded in a weak potential for genetic adaptation to cadmium (Cd), while others reported inheritable increased tolerance in Cd‐contaminated populations. Using quantitative genetics and selection experiments, this study sought to further assess the potential of *Gammarus* populations to genetically adapt to Cd. By combining the control of the reproductive cycle of this species in the laboratory and protocols of individual Cd exposure, we conducted half‐sib analyses to establish the genetic and environmental sources of variance in Cd sensitivity of neonates. Prior to experiments, computations allowed optimizing the experimental design in order to increase the power to detect additive genetic variance. The main findings are the existence of strong between‐brood variability along with weak heritability of Cd sensitivity within *Gammarus* populations. This study also revealed a significant maternal effect on individual Cd sensitivity. This sheds new light on the importance of maternal influence in microevolutionary processes occurring in contaminated environments.

## Introduction

Deciphering evolutionary processes occurring within long‐term contaminated aquatic populations is important for the ecological risk assessment of toxic chemical contaminants (Bickham 2011; Coutellec and Barata 2011; De Coninck et al. 2014). Indeed, if tolerance to contaminants frequently occurs in natural populations exposed to environmental contamination, water quality criteria established from short‐term standardized ecotoxicity tests might be over protective; on the opposite, if tolerance is associated with fitness costs, especially expected in case of genetic adaptation which can lead to a loss of genetic diversity in populations, current values of water quality criteria might be under protective if tolerance frequently arises in contaminated environments (Klerks and Weis 1987; Weis 2002). But despite the fact that evolutionary processes induced by contaminants is deep of concern for a whole understanding of impacts of chemical stressors on the environment, attention from regulators to consider such biological effects in ecological risk assessment (*e.g.,* for the derivation of environmental quality standards) has remained limited (De Coninck et al. 2014). However, the fact that long‐term exposure to a pollutant can induce increased tolerance in aquatic animal populations is now well documented (Johnston 2011). But whether such phenomena of increased tolerance occur through genetic adaptation and/or acclimation processes has not yet been fully elucidated, especially for field studies involving natural populations. Indeed, the examination of transgenerational or evolutionary responses to pollution has most often been experimentally studied in model species suitable for multigeneration exposure in laboratories (Coutellec and Barata 2013). Yet the application of quantitative genetics in an ecotoxicological framework offers an alternative strategy to track the potential of the evolution of tolerance to contaminants within field populations (Klerks et al. 2011). As exemplified with the fish sheepshead minnow (Klerks and Moreau 2001), estimating the heritability of sensitivity based on resemblances among relatives can be operated for individuals originating from natural populations. In the freshwater amphipod *Gammarus*, a highly ecologically relevant species commonly used in aquatic ecotoxicology both in the laboratory and in the field (*e.g.,* reviewed in Kunz et al. 2010), previous work in evolutionary ecotoxicology has focused on the variability of the sensitivity to heavy metals, with contradictory results on the possibility for *Gammarus* populations to adapt to metals in the field (Maltby and Crane 1994; Khan et al. 2011; Boets et al. 2012). In this context, we previously conducted a sib analysis study with *Gammarus fossarum* (Chaumot et al. 2009). This study demonstrated the existence of between‐brood differences in lethal tolerance to Cd, but such differences were not heritable because additive genetic variation was weak. This result predicts that *Gammarus* populations would not likely evolve genetic resistance in a context of persistent Cd pollution. In contrast, we have recently identified a natural population of *G. fossarum* in a stream long‐term contaminated by natural geochemical source of Cd. This population had developed Cd tolerance that is transmissible to offspring, even when genitors were placed in an uncontaminated environment (Vigneron et al. 2015). Moreover, tolerance and associated costs in that population have evolved for water Cd concentrations that are ten times lower than concentrations for which the phenomenon of microevolution in response to contaminants has already been reported (De Coninck et al. 2014). These findings raised questions from an applied point of view, because these concentrations are below the current regulatory protective thresholds of acceptable environmental Cd contamination (*e.g.,* European environmental quality standard).

This recent observation of increased Cd tolerance in field populations of *G. fossarum*, along with the fact that the conclusion on the absence of additive genetic variance in Cd sensitivity in this species can be disputed with regard to the lack of statistical power of the sib design employed (Chaumot et al. 2009), involves to more thoroughly reconsider whether naïve populations of *G. fossarum* have the potential to evolve genetically in response to Cd and thus to better understand the underlying mechanism of Cd tolerance in this species. To this end, this study adopted approaches supplying predictive measurements of the potential to adapt genetically through quantitative genetics. More specifically, considering individuals originating from two naïve populations, we employed approaches described by Klerks et al. (2011) that allow answering two different questions: (i) Is there a response to artificial selection for resistance to Cd? (ii) Is Cd sensitivity influenced by the pedigree of individuals and is it heritable? To answer the first question, we mimicked the first step of a selection process in a short‐term artificial selection experiment with the objective of assessing the possibility of selecting tolerant individuals. To accomplish this task, the sensitivity of offspring from males having survived after a Cd exposure lethal for 80% of the population was compared to the offspring of unexposed genitors. We responded to the second question by means of sib analyses that decomposed the phenotypic variance (*V*_*P*_) of the trait ‘Cd sensitivity’ (individually assessed by survival time of juvenile organisms exposed to Cd) between the different causal components of variance, namely (i) additive genetic variance (*V*_A_), (ii) nonadditive genetic variance, which includes both dominance effects (*V*_D_) and epistatic interactions (*V*_I_), and (iii) environmental variance (*V*_E_) (Falconer and Mackay 1996). The comparison of Cd sensitivity in either full‐sibs or paternal or maternal half‐sibs therefore allowed estimating *V*_A_ and its proportionate phenotypic variance of Cd sensitivity, that is, the narrow sense heritability (*h* ^2^) of Cd sensitivity, as well as a nongenetic maternal component within environmental influences. Two different naïve populations of *G. fossarum* from uncontaminated streams were tested because heritability of a given character is specific to a particular population under particular conditions (Falconer and Mackay 1996). In addition, the experimental design was optimized (number of pairs *vs* number of juveniles tested per brood) by means of computations before experiments, seeking to maximize the statistical power for *V*_*A*_ detection. Overall, beyond the validation of the hypothesis of a weak potential for *Gammarus* populations to genetically adapt to Cd, the experimental design demonstrated an unexpected significant maternal effect on the distribution of individual Cd sensitivity within populations. This result sheds new light on the importance of maternal influence in transgenerational processes underlying evolution of tolerance to contaminants.

## Materials and methods

### Collection of test organisms, maintenance, and experimental conditions

*Gammarus fossarum* adults were collected at an upstream location of the Bourbre River, Isère, France (45°34′09.9ʺN; 05°27′33.9ʺE), a population denoted ‘Tour’, which corresponds to the population used in our previous study (Chaumot et al. 2009). In addition, a second population, ‘Bois’, was selected from at an upstream location in a tributary of the Guiers Mort River, Isère, France (45°24′04.5ʺN; 05°45′36.5ʺE). A previous biomonitoring program demonstrated the absence of bioavailable metallic contamination in these stations, notably for Cd (Besse et al. 2013; Vigneron et al. 2015). *Gammarus fossarum* were kick‐sampled using a hand net and quickly transported to the laboratory in plastic bottles containing ambient freshwater. Before experiments, the organisms were maintained for 2 weeks in 20‐L tanks under constant aeration and continuously supplied with drilled groundwater (conductivity 490 μS cm^−1^), a 16‐h light/8‐h dark photoperiod, and a temperature of 12°C. They were fed *ad libitum* with conditioned alder leaves (*Alnus glutinosa*) and supplied weekly with *Tubifex* larvae.

In exposure conditions, a random spatial distribution of replicates, populations, and/or conditions within experimental systems was applied in all experiments. Cd was provided by Sigma‐Aldrich (St. Quentin Fallavier, France). To assess offspring Cd sensitivity, the sensitivity of juveniles was assessed individually by measuring time during exposure to the lethal level of 20 μg Cd L^−1^, by means of semistatic exposure in 50‐mL polypropylene tubes (BD FalconT). The concentration of 20 μg Cd L^−1^ was determined from Chaumot et al. (2009) with the aim to ensure both sensitivity and specificity to quantify sensitivity to acute Cd stress (*i.e*., not a too high concentration in order to scatter mortalities in time, and not a too long exposure in order to attribute mortalities to Cd). Hence, in previous studies considering *Gammarus* juveniles from the Tour population (Chaumot et al. 2009; Vigneron et al. 2015), 20 μg Cd L^−1^ gave rise to mortalities scattered from 12 h to 10 days of exposure, while only few death occurred in the controls. Survival was monitored every day until all individuals died. The test solution was renewed every 48 h from a common test solution prepared the day of the renewal from a stock solution of 20 mg Cd L^−1^ (CdCl2‐2.5H2O in demineralized water) and with drilled groundwater mixed with osmosed water at constant conductivity (300 μS cm^−1^) and water hardness (141 and 106 mg L^−1^ CaCO_3_ for the short‐term experiment of artificial selection and sib analyses, respectively). For each experiment, juveniles were not fed during exposure and they were exposed at a predetermined age, taking into account their date of release from the maternal brood pouch.

### Short‐term experiment of artificial selection

**Figure 1:**
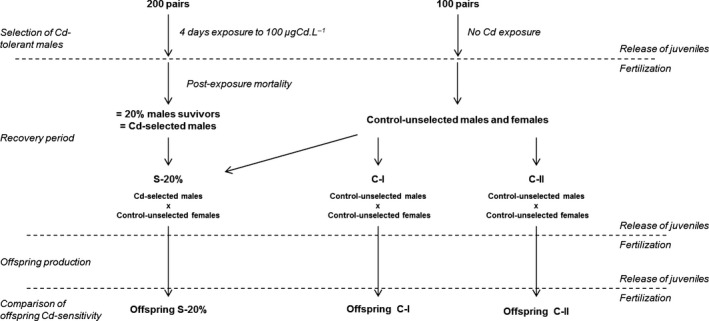
Schematic overview of the artificial selection experiment.

The 20% most Cd‐resistant male genitors within a sample of 200 mating pairs from the Tour population were selected by truncation (Falconer and Mackay 1996), during an acute exposure to Cd (80% mortality including postexposure mortality). A schematic overview of the experimental design for juvenile production is presented in Fig. 1. Two weeks after field sampling, 200 mating pairs in the last stage of their reproductive cycle were isolated [as described in Geffard et al. (2010)] and exposed to 100 μg Cd L^−1^ for 4 days during semistatic exposure, in replicates of ten pairs in 500‐mL polypropylene beakers. The test solution was renewed every 24 h from a common solution of a nominal 100‐μg Cd L^−1^ concentration prepared every 24 h. Animals were not fed during the exposure, and dead animals were removed every day. One hundred additional mating pairs were submitted to the same treatment except for Cd exposure in order to provide control genitors. Replicates were pooled after exposure in 6‐L tanks and maintained under control conditions for approximately 2 months. Spermatogenesis, embryogenesis dynamics, and female reproductive cycle are fully described in *G. fossarum* (Geffard et al. 2010; Trapp et al. 2015), allowing the controlled production of offspring. Hence, during the first 2 weeks, juveniles present in marsupium were released by females, and the first fertilization and egg laying occurred. At this stage, the juveniles released were removed from all conditions, and females in Cd‐exposed treatment were also discarded. Afterward, three sets of genitors denoted S‐20%, C‐I, and C‐II, were constituted: S‐20% grouped males of Cd‐selected individuals mated with unexposed females; two replicates of the control condition (C‐I and C‐II) were formed with both unexposed males and females. An equal number of mating pairs were respected for each condition, and the two replicates of reduced control (C‐I and C‐II) were used to take into account the effect of a potential random drift due to the restricted number of Cd‐selected individuals. One month later, the first set of juveniles produced from the first egg fertilization were released from the marsupium of females and removed from the experiment. A new fertilization by males in S‐20%, C‐I, and C‐II conditions occurred. One month later again, the release from the females' marsupium was monitored to obtain the juveniles produced from this second fertilization for Cd sensitivity assessment. This design guarantees that the juveniles tested did not experience any environmental direct or indirect influence of Cd exposure (male and female gametogenesis in clean conditions, no maternal exposure). The choice has been made to assess whether the selection is efficient on the next generation, by measuring Cd sensitivities of offspring at the juvenile stage rather than in adults to avoid fitness alteration unrelated with toxicological sensitivities (*e.g*., selection of individuals resistant to laboratory conditions) that could arise during the rearing of juveniles in the laboratory until they reach sexual maturity (approximately 5 months at 12°C; Coulaud et al. 2014). Offspring were therefore exposed at the juvenile stage, from 1 to 7 days old, to a lethal concentration of 20 μg Cd L^−1^. A sample of offspring was also used as control of survival in clean conditions. Exposure levels to Cd for adult males and juveniles have been designed to make the measure of the Cd sensitivity of these two different stages comparable in terms of level of acute toxicity. Hence, 100 μg Cd L^−1^ and 20 μg Cd L^−1^ were selected because they led to equivalent LT50s in previous studies with *Gammarus* from the Tour population (Chaumot et al. 2009; Vigneron et al. 2015).

### Breeding design for sib analysis and estimation of genetic parameters

The same breeding protocol as described in Chaumot et al. (2009) was used. Briefly, *G. fossarum* adults were collected within both the Tour and Bois populations. Two weeks after field sampling, mating pairs with females synchronized in the last stage of their reproductive cycle were isolated until they produced three successive broods in individual 500‐mL glass beakers (for approximately 2 months). The first batch of juveniles (obtained 1 week after the selection of mating pairs) was constituted by individuals with unknown fathers (fertilization in the field before sampling), and the second batch (released 1 month later) by the offspring of the guarding males present when pairs were selected. After the first fertilization of females, males were randomly redistributed between beakers, so that females would be fertilized by a different male for the third brood. Thus, at the end, each female produced juveniles from three different males, and each male identified provided offspring from two different females. This controlled mating scheme gave rise to three batches of broods for each population yielding both paternal and maternal half‐siblings. Juveniles were exposed to 20 μg Cd L^−1^ beginning the day following their release from the maternal brood pouch. The same protocol as described for the offspring in the artificial selection experiment was followed.

As detailed in Table S1, the causal components of phenotypic variance of Cd sensitivity (*V*_*P*_) can be related to the observational components of variance in the different sib designs (Falconer and Mackay (1996); Lynch and Walsh (1998)), namely $\sigma_{B-\mathrm{FS}}$ and *σ*_R‐FS_ the ‘brood’ and residual variance components, respectively, in the full‐sib designs; *σ*_S‐PHS_, *σ*_D‐PHS_, and *σ*_R‐PHS_ the ‘sire’, ‘dam within sire’, and residual variance components, respectively, in the paternal half‐sib design; and finally, *σ*_D‐MHS_, *σ*_S‐MHS_, and *σ*_R‐MHS_ the ‘dam’, ‘sire‐within‐dam’, and residual variance components, respectively, in the maternal half‐sib design. Classically, we assumed that nonadditive genetic variances are limited to dominance genetic variance *V*_D_. With this experimental design (*e.g.,* individual exposure), common environment influences are restricted to the marsupial incubation period. Therefore, beside a residual environmental variance (*V*_Ew_), we distinguished two components in the common environment variance (*V*_Ec_): a maternal effect (*V*_Em_) (nongenetic influence of the mothers, which enhances the resemblance between offspring that share the same mother, whatever brood they belong to) and a brood‐specific maternal effect (*V*_Esm_) (nongenetic influence of the mothers, which enhances the resemblance of the sibs of a given brood but is unrepeatable for successive broods).

Heritability was estimated from both the paternal and the maternal designs, from sire and dam components, respectively, as follows:

(1) $$h_{\mathrm{PHS}}^{2}=4\frac{\sigma_{S-\mathrm{PHS}}^{2}}{V_{P}}=\frac{V_{A}}{V_{P}}$$

(2) $$h_{\mathrm{MHS}}^{2}=4\frac{\sigma_{D-\mathrm{MHS}}^{2}}{V_{P}}=\frac{V_{A}}{V_{P}}+4\frac{V_{\mathrm{Em}}}{V_{P}}$$

The degree of similarity between sibs was also quantified by means of intraclass correlation coefficients. Hence, full‐sib correlations were calculated from the three designs as follows:

(3) $$t_{\mathrm{FS}}=\frac{\sigma_{B-\mathrm{FS}}^{2}}{V_{P}}=\frac{1/2V_{A}+1/4V_{D}+V_{\mathrm{Em}}+V_{\mathrm{Esm}}}{V_{P}}$$

(4) $$t_{\mathrm{FS}-\mathrm{PHS}}=\frac{\sigma_{S-\mathrm{PHS}}^{2}+\sigma_{D-\mathrm{PHS}}^{2}}{V_{P}}=\frac{1/2V_{A}+1/4V_{D}+V_{\mathrm{Em}}+V_{\mathrm{Esm}}}{V_{P}}$$

(5) $$t_{\mathrm{FS}-\mathrm{MHS}}=\frac{\sigma_{D-\mathrm{MHS}}^{2}+\sigma_{S-\mathrm{MHS}}^{2}}{V_{P}}=\frac{1/2V_{A}+1/4V_{D}+V_{\mathrm{Em}}+V_{\mathrm{Esm}}}{V_{P}},$$

from the full‐sib, the paternal half‐sib, and the maternal half‐sib designs, respectively. Paternal and maternal half‐sib correlations were also calculated from the paternal half‐sib and the maternal half‐sib designs, respectively, as follows:

(6) $$t_{\mathrm{PHS}}=\frac{\sigma_{S-\mathrm{PHS}}^{2}}{V_{P}}=\frac{1/4V_{A}}{V_{P}}$$

(7) $$t_{\mathrm{MHS}}=\frac{\sigma_{D-\mathrm{MHS}}^{2}}{V_{P}}=\frac{1/4V_{A}+V_{\mathrm{Em}}}{V_{P}}$$

Standard errors of heritability estimates and intraclass correlation coefficients were calculated as described by Lynch and Walsh (1998) for balanced designs.

### Computations and statistical analyses

All statistical procedures and computations were carried out with the R software (R Development Core Team 2012).

#### Sib design optimization

The experimental design for sib analyses was optimized based on the estimates of variance components of Cd sensitivity from the previous study conducted with the Tour population (Chaumot et al. 2009). To enhance the statistical power for detecting additive genetic variance, and thus heritability, we first aimed to maximize the number of broods tested per batch, by minimizing the number of sibs tested per brood. We therefore simulated 100 balanced full‐sib designs respecting brood and residual variance estimated by Chaumot et al. (2009) with different numbers of broods (from 10 to 100). Based on the 95% confidence interval, we assessed how the precision of the estimation of the between‐brood variability (*σ*_B‐FS_) was affected by the reduction in the number of sibs tested per brood (from ten to three). This allowed setting the number of juveniles tested per brood to conduct further experiments. Thereafter, we similarly assessed the precision of the estimate of the sire effect (*σ*_S‐PHS_) in the paternal half‐sib design for different numbers of sires tested considering different values of *h* ^2^. The distribution of *σ*_S‐PHS_ estimates was established from 100 simulated datasets of paternal designs considering two mated females per male, respecting interbrood and residual variance estimated by Chaumot et al. (2009), for different numbers of sires (10, 20, 30, 40, 50, 100, 200, 400) and for different values of theoretical *h* ^*2*^ (0.1, 0.2, 0.4, 0.6, 0.8).

#### Statistical inference

As in Chaumot et al. (2009), the observational variance components of Cd sensitivity of full‐sibs and paternal and maternal half‐sibs were estimated using variance decomposition of log‐transformed survival times using linear mixed‐effect models. Restricted maximum‐likelihood (REML) estimators and 95% confidence intervals were computed with the lme function from the nlme library within the R software. Significance of random effects (brood, sire, dam) was assessed by comparison of models through likelihood ratio tests (LRT), and the significance of fixed effects (batch) was tested by anova (Pinheiro and Bates 2000). AIC and BIC criteria were also inspected, but they are not presented here because they always agreed with LRT. Hence, we tested for full‐sibs the existence of differences in Cd sensitivity between broods (*i.e.,* random ‘brood effect’) for the three batches of each population separately. The model without a brood effect was fitted using the gls function from the nlme library using the REML method. For the paternal (respectively, maternal) half‐sib design, we built a model considering a random sire (respectively, dam) effect, a random dam effect nested within the sire effect (respectively, sire effect within the dam effect), and a residual environmental effect. The significance of the sire (respectively, dam) effect was assessed by LRT between the full model and a nested model considering only one random brood effect. The possibility of an effect induced by the fact that three batches of broods were exposed to Cd at three different dates was assessed by testing the significance of a fixed ‘batch effect’ on Cd sensitivity. As this ‘batch effect’ appeared to be significant, it as has been included as a fixed effect in mixed‐effect models for all half‐sib analyses.

## Results

### Selection of male genitors did not lead to tolerant offspring

**Figure 2:**
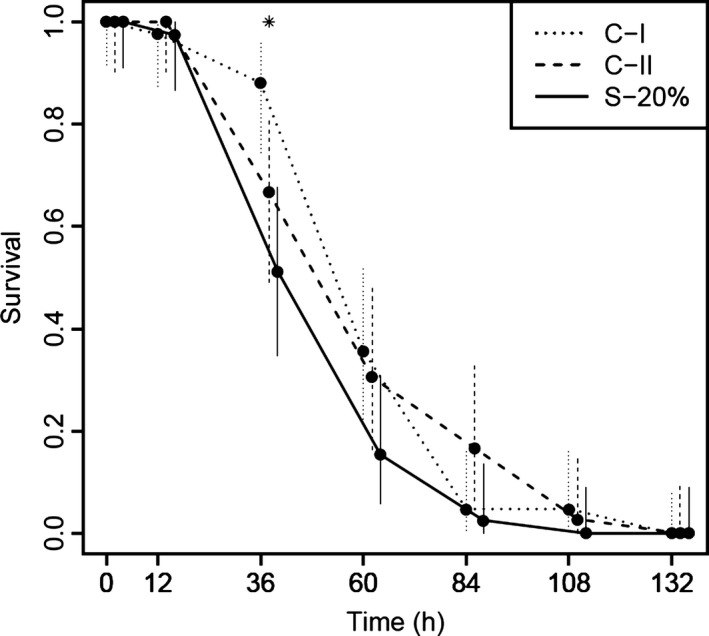
Artificial selection experiment. Dots represent the mean survival rates recorded for offspring from the selected and the two control conditions (S‐20%, C‐I, C‐II) exposed to 20 μg Cd L^−1^. Binomial 95% confidence intervals are pictured by vertical lines. Asterisk indicates time for which significant difference has been detected between the three conditions.

In the artificial selection experiment, we aimed to select the 20% most Cd‐tolerant male genitors within the Tour population. The Cd sensitivity of the juveniles produced by these individuals when mated for their second fertilization after Cd exposure with naïve females (*i.e.,* spermatogenesis in clean conditions) was compared to control offspring from unselected males. After the first fertilization event (16 days after the end of Cd exposure), the survival of Cd‐selected males was equal to 19.5%. After the second fertilization event (58 days after the end of Cd exposure), it was only equal to 12.5%. This corresponds to 64% survival between the two copulations, whereas it exceeded 85% for unselected control males. This postexposure mortality thus implied that the percentage of males selected for their Cd resistance was not 20% as expected, but between 12.5% and 19.5%. Nonetheless, postexposure mortality was rapidly stabilized after the first fertilization event (13% survival at 24 days after the end of Cd exposure). Finally, 42, 36, and 39 juveniles from the second batch of broods were exposed to 20 μg Cd L^−1^ for conditions C‐I, C‐II, and S‐20%, respectively, and 28, 20, and 26 juveniles were used as controls (*i.e*., not exposed to Cd) for the three conditions, respectively. All mortalities occurred in exposed juveniles within 6 days of Cd exposure, and no mortality was observed in juveniles used as control during this time. Time–response curves (Fig. 2) are very similar for the three conditions. Nonetheless, a comparison of the survival rate at each date brought out a significant difference only at one time between the three conditions (prop.test: *P *=* *0.002 at 36 h; Fig. 2). As only one of the two control sets of exposed juveniles deviated from the S‐20% juveniles, this difference observed at one date is more likely attributable to variability due to random sampling because of a small number of males and females (16 mating pairs per condition) used to produce juveniles. In addition, the juveniles of the S‐20% Cd‐selected condition did not have a greater LT50 in comparison with control conditions (36.8 h vs 53.4 h for C‐I and 47.1 h for C‐II).

### Sib analyses of Cd sensitivity revealed between‐brood variability, low heritability, and maternal effect

#### Breeding design optimization

**Figure 3:**
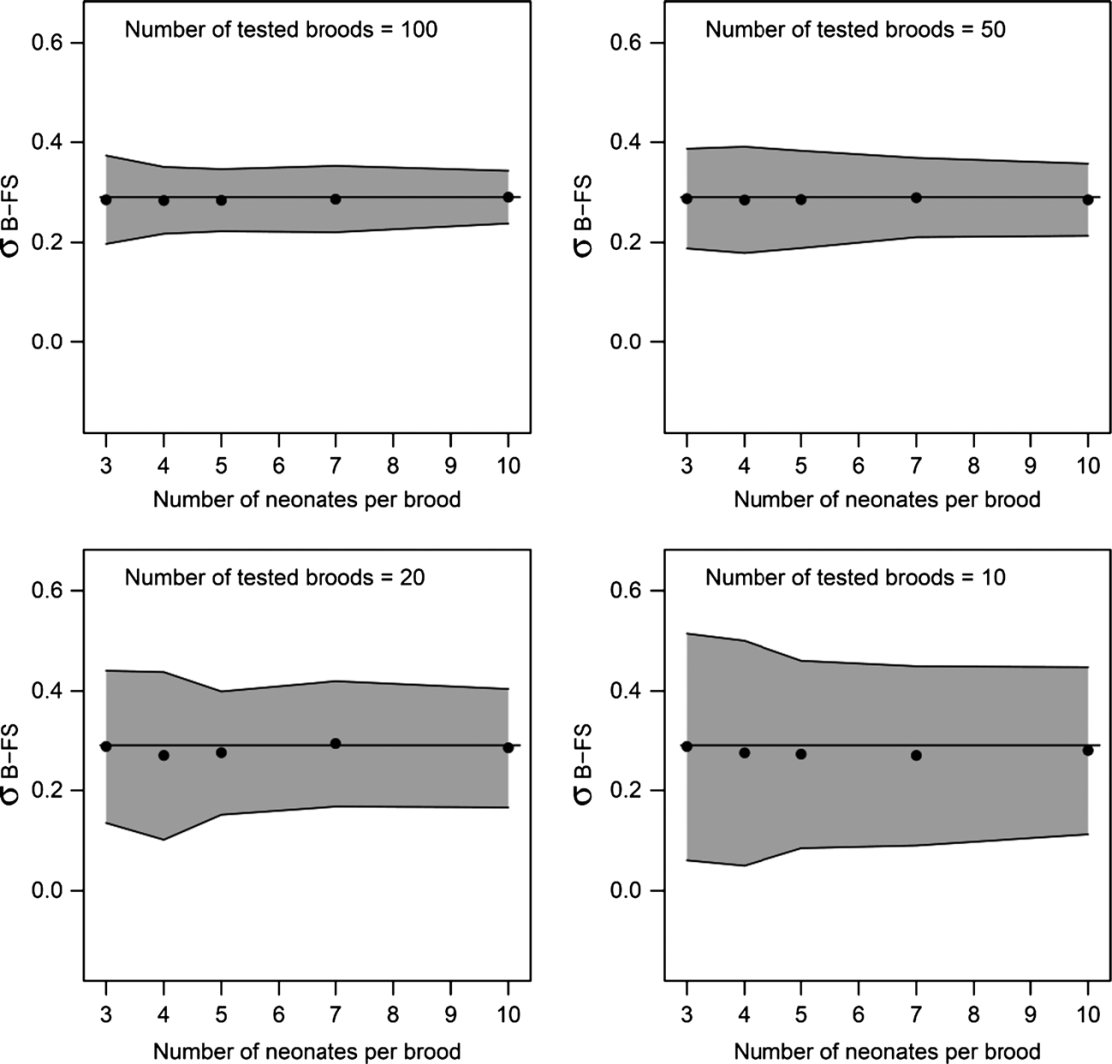
Estimation of brood component and optimization of the experimental design. 95% confidence intervals of the estimate of the brood component (*σ*_B‐FS_) were simulated for different number of broods (10, 20, 50, 100) and for different number of neonates tested per brood (3, 4, 5, 7, 10), based on brood and total variances estimated by Chaumot et al. (2009). Dots represent average *σ*_B‐FS_ calculated from 100 simulations. Solid lines represent expected value.

**Figure 4:**
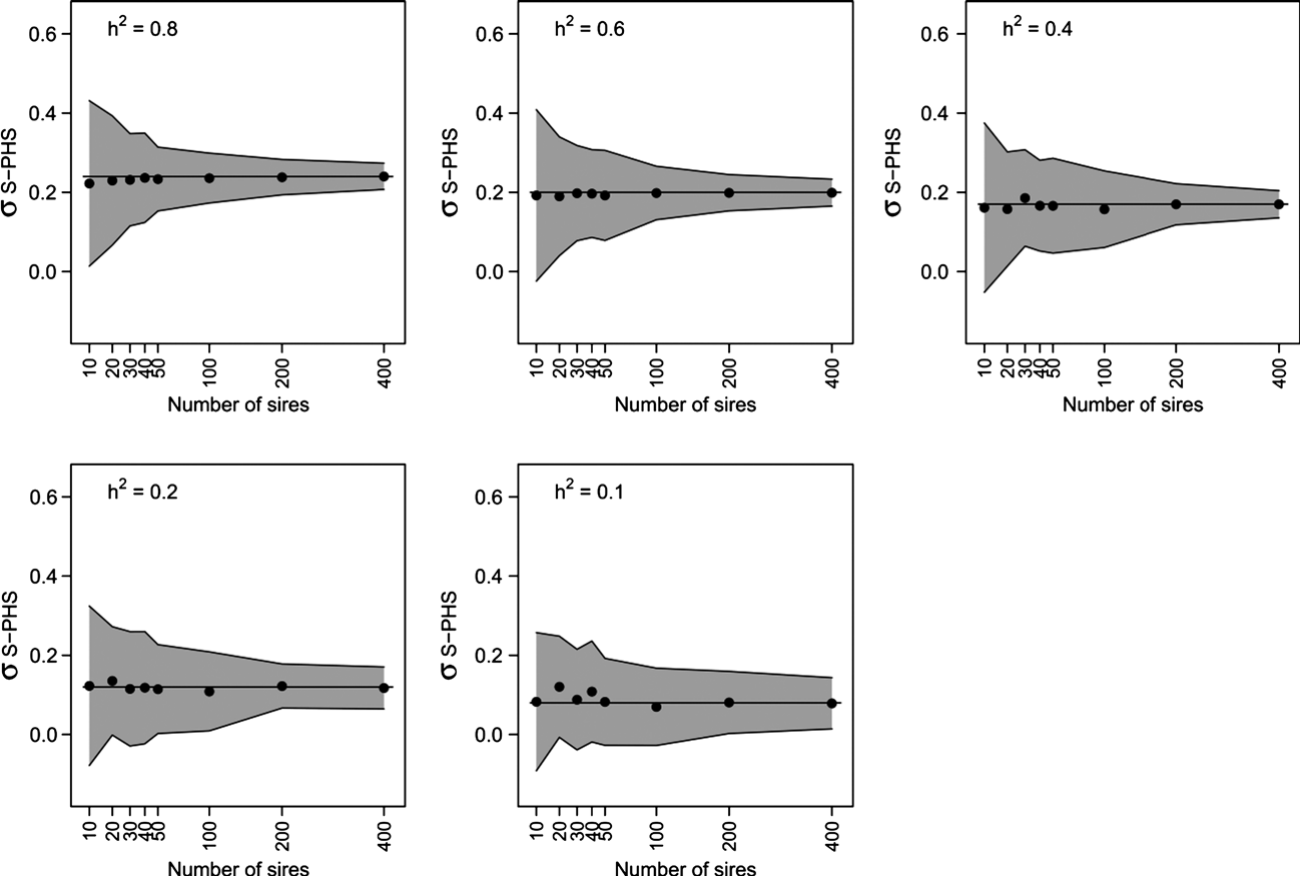
Estimation of the sire component and optimization of the paternal design. 95% confidence intervals of the estimate of the sire component (*σ*_S‐PHS_) were simulated for different values of *h* ^*2*^ (0.1, 0.2, 0.4, 0.6, 0.8) and for different number of sires tested (10, 20, 30, 40, 50, 100, 200, 400), based on brood and total variances estimated by Chaumot et al. (2009). Dots represent average *σ*_S‐PHS_ calculated from 100 simulations. Solid lines represent expected values.

The analysis of the 100 simulated full‐sib datasets based on the variance components of Cd sensitivity established by Chaumot et al. (2009) indicated that the precision of the *σ*_B‐FS_ REML estimator was poorly improved by increasing the number of juveniles tested per brood beyond five individuals whatever the number of broods tested (Fig. 3). This is why, contrary to our previous study, in a balanced design we exposed only five juveniles for all broods, discarding possible additional released juveniles from the experiment. This allowed testing more broods per batch and population, which increases the precision of *σ*_B‐FS_ estimation (Fig. 3). Furthermore, the analysis of the 100 simulated paternal half‐sib datasets (five juveniles per brood, two dams per sire), assuming different theoretical values for *h* ^2^ (from 0.1 to 0.8), indicated that the precision of the *σ*_S‐PHS_ REML estimator (and therefore *h* ^2^ detection) was substantially improved for *h* ^2^ values above 0.2 when the number of tested sires increased from 10 to 30 and to a lesser extent from 30 to 200 (Fig. 4). The precision of *σ*_S‐PHS_ estimation remained only slightly improved by increasing the experimental effort (number of sires) for low values of *h* ^2^ (here 0.1). From these findings, experiments were designed seeking to test at least 30 sires in paternal half‐sib designs. Overall, the survival of 713 neonates was individually monitored for the Tour population and 442 for the Bois population. For most of the broods, we obtained five juveniles, but incomplete broods were still included in the analysis because mixed‐effect models make it possible to analyze such unbalanced data (Pinheiro and Bates 2000).

#### Full‐sibs

Substantial differences in tolerance to Cd were observed between broods for the two populations. Median lethal times of broods for the first batch ranged from 0.5 days to 5.5 days for the Tour population and from 0.5 to 9.5 days for the Bois population. For the second and third batches of broods, median lethal times of broods for the population Tour ranged from 1.5 to 13.5 days and from 0.5 to 7.5 days respectively, and from 0.5 to 11.5 days and from 1.5 to 8.0 days, respectively, for the population Bois. This brood effect (Table 1) was significant for the three batches for the Tour population and only for the first two batches for the Bois population (LRT: *P *<* *0.05). No significant brood effect was detected for the third Bois population batch (LRT: *P *=* *0.29), along with a large 95% confidence interval associated with *σ*_B‐FS_ (Table 1). This lack of precision of the *σ*_B‐FS_ estimator is due to the low number of broods tested and the presence of incomplete broods. The degree of similarity between full‐sibs, assessed with the value of *t*_FS_ (Table 1), is similar for the two populations for the first batch (between 25 and 30%). This indicator of full‐sib resemblance is constant for the second Tour batch. Fluctuations were observed for other batches, *that is,* an increase for the second Bois batch and decreases for the third batches of both populations. However, these values are associated with less accurate estimations of *σ*_B‐FS_.

**Table 1:** Full‐sib analysis of the Cd sensitivity of neonates from the two populations. Here are provided REML estimators (and 95% confidence intervals) of the variance components from a linear mixed‐effect model considering log‐transformed lethal time of neonates as a response explained by a brood effect (*σ*_B‐FS_) and a residual environmental variance (*σ*_R‐FS_)

Population	RE ML estimator		n broods (n neonates)	*t*_FS_
	*σ*_B‐FS_	*σ*_R‐FS_		
Tour				
1st batch of broods	0.40 [0.30, 0.54]**	0.64 [0.58, 0.70]	53 (265)	0.28
2nd batch of broods	0.37 [0.28, 0.49]**	0.54 [0.50, 0.60]	53 (265)	0.31
3rd batch of broods	0.22 [0.11, 0.45]*	0.66 [0.59, 0.74]	38 (183)	0.10
Bois				
1st batch of broods	0.43 [0.31, 0.60]**	0.72 [0.64, 0.80]	62 (237)	0.26
2nd batch of broods	0.61 [0.46, 0.82]**	0.60 [0.53, 0.68]	34 (159)	0.51
3rd batch of broods	0.18 [0.02,1.37]^NS^	0.63 [0.50, 0.80]	11 (46)	0.07

The significance of the brood effect was tested by LRT (NS not significant, * for *P *<* *0.05 and ** for *P *<* *0.01).

#### Batch effect

For both the Tour and Bois populations, linear mixed‐effect modeling of Cd sensitivity detected a significant fixed batch effect (anova:* P *< 10^−4^ and *P *=* *0.0046 for the Bois and Tour populations, respectively), but no significant interaction between batch effect and the between‐brood variability (LRT: *P *=* *0.6603 and *P *=* *0.387 for the Bois and Tour populations, respectively). This indicates that there was a variation in the average level of Cd sensitivity between batches, but that the between‐brood variability remained unchanged from the first to the third batch of broods. Therefore, half‐sib analyses were performed including a fixed batch effect in the mixed‐effect models. It should be noted that the median lethal time for juveniles did not change monotonously from the first to the third batch (mean lethal time from the first to the third batch, 2.8, 4.1, and 3.1 for the Tour population and 4.8, 3.2, and 3.7 for the Bois population). This disqualifies any hypothesis of uncontrolled selection or depletion of genitors in laboratory conditions, which could induce increasing or decreasing Cd sensitivity in their offspring during the 3 months of the experiment.

#### Half‐sibs

For the Tour population, the survival of 373 neonates was recorded in the paternal half‐sib design, coming from 76 broods and resulting from the successful mating of 38 sires with two different dams. The analysis of distribution of paternal half‐sib sensitivity did not detect any significant sire effect (Table 2) (LRT: *P *=* *0.818). This is also reflected by the low *t*_PHS_ and $h_{\mathrm{PHS}}^{2}$ values (Table 2). Moreover, considering formulas (4) and (6), the fact that *t*_FS−PHS_ (0.236) is more than twice *t*_PHS_ (0.015) suggests that dominance (*V*_*D*_) and/or maternal effects (*V*_Em_ and *V*_Esm_) contribute to the resemblance between full‐sibs. This substantial contribution of dominance and/or maternal effects is also shown by the significant dam within sire component ($\sigma_{D-\mathrm{PHS}}$), which is much greater than the sire component (Table 2). Concerning the maternal half‐sib design, 38 dams were successfully mated with three different sires for the Tour population and the survival of 563 neonates was recorded. For this population, a significant dam effect was detected (LRT: *P *=* *0.01) (Table 2). The dam component ($\sigma_{D-\mathrm{PHS}}$) estimate is equal to 0.23 and leads to a $h_{\mathrm{MHS}}^{2}$ of 0.417 (±0.190) and a *t*_MHS_ of 0.104 (±0.047). This higher resemblance between maternal half‐sibs than paternal half‐sibs (*t*_MHS_ >> *t*_PHS_) suggests that maternal effects are a significant source of variation. Furthermore, the sire‐within‐dam component estimate is large (0.28), with a relatively low 95% confidence interval, which, when *V*_A_ is null, indicates that *V*_D_ and/or *V*_Esm_ also contribute substantially to the total variance (Table 2), corroborating the findings from the paternal design.

**Table 2:** Half‐sib analysis of the Cd sensitivity of neonates from the Tour population, considering paternal and maternal designs

Observational variance component		REML estimator [IC 95%]	Sib correlations ± SD	Estimates of heritability ± SD
		Tour		
Paternal design		38 sires nested with 2 dams		
Sires	*σ*_S‐PHS_	0.08^NS^ [0.00; 6.01]	*t*_FS‐PHS_ 0.24 ± 0.08	$h_{\mathrm{PHS}}^{2}$ 0.06 ± 0.25
Dams within sires	*σ*_D‐PHS_	0.32 [0.22; 0.48]	*t*_PHS_ 0.01 ± 0.06	
Residual	*σ*_R‐PHS_	0.60 [0.55; 0.65]		
Maternal design		38 dams nested with 3 aires		
Dams	*σ*_D‐MHS_	0.23* [0.14; 0.37]	*t*_FS‐MHS_ 0.26 ± 0.05	$h_{\mathrm{MHS}}^{2}$0.42 ± 0.19
Sires within dams	*σ*_S‐MHS_	0.28 [0.21; 0.39]	*t*_MHS_ 0.10 ± 0.05	
Residual	*σ*_R‐MHS_	0.61 [0.57; 0.65]		

SD, standard deviation.

Here are provided REML estimators (and 95% confidence intervals) of the observational components of variance with significance tested by LRT (* for *P *<* *0.05), along with estimated genetic parameters.

Overall, we conclude that sensitivity to Cd is not heritable in the Tour population, because the contribution of additive genetic variance to offspring Cd sensitivity determinism is weak and because this trait is governed by maternal effects and potentially by nonadditive genetic components (dominance). Unfortunately, the genitors from the Bois population were difficult to maintain under laboratory conditions, and some mortality arose during the experiment, resulting in half‐sib designs with a weak statistical power. Indeed, only seven sires were successfully mated with two different dams, and only ten dams were successfully mated with three different sires. Not surprisingly, the analysis of distribution of offspring Cd sensitivity failed to detect either a significant sire effect or a significant dam effect (Table S2), comparable to what was obtained by Chaumot et al. (2009).

## Discussion

In the artificial selection experiments, we aimed to select the 20% most Cd‐tolerant individuals within the Tour population, but the implementation of the protocol resulted in the selection of a smaller fraction of the population (likely closer to 10%), thus resulting in a higher selection intensity than expected. Only selected males were used and not females, to avoid the influence of a nongenetic transgenerational effect of maternal Cd exposure that could bias the evaluation of the response to genetic selection. For the same reason, we tested only the offspring produced by the second fertilization operated by males after their exposure, thus resulting from gametes produced in clean conditions. All of the juveniles used as controls during Cd tests survived for the three conditions, including the S‐20% juveniles. This indicates that direct effects of parent Cd exposure on juvenile fitness were avoided because of the protocol developed. In these conditions, we observed no elevated resistance to Cd in S‐20% juveniles, and so selection for Cd resistance in the Tour population appeared inoperative, at least in the first generation. This negative result is not conclusive *per se*. Indeed, false‐negative detection could arise because only males were selected, potentially leading to a dilution of the frequency of resistance genes by the influx of susceptible alleles from unselected females (Hoffmann and Hercus 2000; Woods and Hoffmann 2000) or because we tested only one generation. Nevertheless, in an artificial selection experiment for Cd resistance, Xie and Klerks (2003) reported an increased resistance to Cd in the least killifish (*Heterandria formosa*) after only one generation of selection. These authors reported this rapid response for two of three selection lines, while the third selection line only showed an elevated resistance to Cd in the second generation. A false‐negative case could also be that the number of individuals on which we applied the selection pressure (200 males) may have been insufficiently large to contain the rare resistance alleles from the field population in case of a major gene determinism of Cd sensitivity (Woods and Hoffmann 2000). Nevertheless, it appeared from the full‐sib designs for both the Tour and Bois populations that a strong variability of Cd sensitivity can be generated even with only 50 pairs of genitors (ratio of ten between the broods’ minimum and maximum median lethal times) and thus that our sample size of 200 males was sufficient. Because of these interpretation limitations, the absence of an observed response to selection is not fully conclusive by itself in terms of the possibility for a naïve *G. fossarum* population to evolve genetic resistance to Cd, but this result was corroborated by the study's quantitative genetics assay.

Indeed, sib analyses revealed that the heritability in the narrow sense of Cd sensitivity was nonsignificant in both the Tour and Bois populations, leading us to conclude that these populations would not have the potential to adapt genetically to Cd. This reiterates previous findings by Chaumot et al. (2009) for the Tour population. As in this previous study, the absence of paternal half‐sib resemblance contrasts with the strong similarity between full‐sibs. For instance, 28% and 26% of the total phenotypic variance of the first batches of broods for the Tour and Bois populations, respectively, are explained by brood similarity (Table 1). Compared to Chaumot et al. (2009), the statistical power of the experimental design for heritability detection was improved for the Tour population. Here, 38 sires were tested. With this population size, simulations (Fig. 4) indicate that the low *σ*_S‐PHS_ value estimated here for the Tour population is unlikely to be observed (*i.e.,* outside of the 95% confidence interval for simulations) for heritability values >0.2. Therefore, we can conclude that heritability in the Tour population is weak if not null. Furthermore, it appears that a substantially higher number of families would have been needed to detect a low sire component if present. Indeed, as calculated from the simulations for heritability equal to 0.1 and for a design including 400 sires (Fig. 4), a significant sire component would have been detected in only 31% of cases. Thus, the number of sires required would have been prohibitively large, because the nested design used here requires a considerable amount of experimental effort and space. This last point is a weakness of the quantitative genetics approach (Klerks et al. 2011). This failure to detect low values of heritability has also been reported by Klerks and Moreau (2001) and reviewed in Klerks et al. (2011). A possible alternative improvement of the paternal design in *Gammarus* would consist in increasing the number of dams per sire, and this could be feasible in shorter experiments than in the present study because the spermatogenesis cycle is completed in only 7 days at 12°C in this species (Trapp et al. 2015).

In a more detailed analysis, considering that any common environmental effect was excluded as a result of the experimental protocol (individual exposure, randomized design, a single Cd solution, 1‐day‐old neonates), the substantial similarity recorded between full‐sibs could only be explained by shared nonadditive genetic features or by maternal effects (during egg production and brooding). Hence, we detected a significant dam component in the maternal half‐sib design that was not recorded in Chaumot et al. (2009), indicating that the design optimization was effective. The combined use of the two nested designs provided additional information compared to a simple paternal design. It has allowed us not only to show that the heritability of Cd offspring sensitivity was low, but also that maternal effects (*V*_Em_) as well as dominance (*V*_*D*_) and/or a brood‐specific maternal effect (*V*_Esm_) were substantial. As classically applied to data obtained from diallel analyses (detailed in Dawson 1965, Lynch and Walsh 1998), the results of two designs can be combined. The comparison of $h_{\mathrm{PHS}}^{2}$ and $h_{\mathrm{MHS}}^{2}$ estimators makes it possible to quantify *V*_Em_ (maternal effect repeatable between the successive broods of a mother), which accounts for 9% of the total variance of juvenile Cd sensitivity. Despite the fact that a significant maternal effect has been revealed, the residual variance *V*_*Ew*_ appeared to be quite high. As exposure conditions and organisms were well standardized, this variance is unlikely explained by environmental variance. This high residual variance rather results from stochasticity in death event during exposure. As reviewed by Ashauer and Brown (2008), the assumption that death is stochastic is classical in survival modeling in ecotoxicology ((hazard models). Such stochastic variability of death can be seen in the results of toxicity test involving clones. For instance, Barata et al. (1998) tested the sensitivity of four different clones of *Daphnia magna* to different contaminants and in different environments. The variability among clonal individuals appeared non‐negligible within a single condition, for example*,* the 48 h LC50 of a given clone exposed to Cd range from 174 to 279 μg L^−1^. The same significant residual variance was also present in the result of a quantitative genetics experiment on Cd sensitivity conducted with clones of *Daphnia* (Messiaen et al. 2012).

Overall, this study suggests that genetic adaptation to acute Cd stress seems unlikely to occur within naïve *G. fossarum* populations, because we did not observe a response to selection for Cd resistance, and because we confirmed that the heritability of Cd sensitivity was low. Nonetheless, these levels of acute Cd exposure tested in our study (20 μg L^−1^) can only be recorded in hot spots of metallic contamination, such as rivers impacted by mine drainages, *for example,* 15 μg L^−1^ in the Lot watershed (Achard et al. 2004), 50 μg L^−1^ in the Amous watershed (Cosiot et al. 2009), and 28 μg L^−1^ in the Guadiana River basin (Lopes et al. 2005). Thus, even if acute stress occurs in ecosystems at concentrations quite lower than these extreme cases due to longer exposure, our conclusion should be considered with caution before being extrapolated to contaminated environments, especially when encountered Cd levels only induce chronic and sublethal toxicity. Indeed, in such cases, mechanisms of tolerance different from those involved in response to acute toxic stress could take place in natural populations. In our study, the trait ‘survival to Cd exposure’ has been examined but other fitness traits can also supported differences in Cd sensitivity when chronic toxicity is acting. For instance, adverse effects on reproduction have been observed in *G. fossarum* for Cd concentration of 3 μg L^−1^ (Geffard et al. 2010), and Cd is known to disrupt vitellogenesis in Crustaceans (Yang et al. 2015). Nonetheless, Messiaen et al. (2012) scrutinized the potential for adaptation to Cd in a natural *Daphnia magna* population by studying the heritability of the net reproductive rate under that order of magnitude of Cd stress (3.7 μg L^−1^). This led to relatively low estimations of *h* ^*2*^ (0.23 and 0.06 depending on the temperature). Hence, despite the fact that our findings should not be abusively generalized to environmental conditions, they are in line with the fact that tolerance to contaminants of field populations cannot result solely from genetic adaptation, but also stems from acclimation (*i.e.,* plastic changes) (Klerks and Weis 1987; Morgan et al. 2007) and with the observation that genetic adaptation to contaminants is infrequent (Woods and Hoffmann 2000; Klerks 2002; Millward and Klerks 2002). Hence, the hypothesis of physiological acclimation or the hypothesis of adaptation via rare alleles of major genes would both be admissible hypotheses to reconcile the recorded low heritability of Cd sensitivity in naïve *Gammarus* populations and the fact that field *Gammarus* populations tolerant to Cd are still present in contaminated environments. For instance, we have recently identified a natural population of *G. fossarum* in a long‐term Cd‐contaminated stream, presenting Cd tolerance, which is transmissible to offspring in an uncontaminated environment (Vigneron et al. 2015). In this context, the present finding of a significant dam effect on Cd sensitivity sheds new light on the importance of maternal influence in transgenerational processes underlying the evolution of Cd tolerance in *Gammarus*. Indeed, genetic adaptation, despite low heritability of Cd offspring sensitivity and the absence of a response to selection, could take place through the selection of traits other than tolerance to the toxicant itself such as maternal effects (Gotthard and Nylin 1995; Marshall and Uller 2007). We are therefore planning to investigate the inheritance of Cd tolerance in the identified tolerant field population further. Determining the mechanisms supporting increased tolerance and potentially the inheritance of modified sensitivities in such a case study of field‐exposed populations is needed to assess whether microevolutionary processes and transgenerational effects should be considered for the assessment of the ecological hazard of chemical contaminations toward aquatic environments.

## Data archiving statement

Data available from the Dryad Digital Repository: http://dx.doi.org/10.5061/dryad.ft511

## Supporting information


**Table S1** Relationships between observational and causal components of variance for each kind of relatives.
**Table S2** Results for the half‐sib analysis for the population Bois, REML estimators (NS: not significant LRT).
