# Supplementary material for: Mothers and not genes determine inherited differences in cadmium sensitivities within unexposed populations of the freshwater crustacean Gammarus fossarum
Source: Evol Appl. 2015 Oct 28;9(2):355–66. doi: 10.1111/eva.12327 (PMC4721071; doi:10.1111/eva.12327)
Supplement: Supplementary file 1 — Table S1 Relationships between observational and causal components of variance for each kind of relatives. Table S2 Results for the half‐sib analysis for the population Bois, REML estimators (NS: not significant LRT). [file EVA-9-355-s001.docx]

**Supplementary materials:**

**Table S1.** Relationships between observational and causal components of variance for each kind of relatives. $V_{A}$ = additive genetic variance, $V_{D}$ = dominance genetic variance, $V_{Em}$ = maternal effects, $V_{Esm}$ = brood-specific maternal effect, $V_{Ew}$ *=* residual environmental variance.

**Table S2.** Results for the half-sib analysis for the population Bois, REML estimators (NS: not significant LRT).
